# Gluteal Hydatid Cyst: A Case Report

**Published:** 2019

**Authors:** Mirsalim SEYEDSADEGHI, Jaffar GHOBADI, Negin HAGHSHENAS, Afshin HABIBZADEH

**Affiliations:** 1. Department of Surgery, School of Medicine, Ardabil University of Medical Sciences, Ardabil, Iran; 2. Department of Emergency Medicine, School of Medicine, Ardabil University of Medical Sciences, Ardabil, Iran; 3. Department of Anesthesiology, School of Medicine, Ardabil University of Medical Sciences, Ardabil, Iran; 4. Department of Internal Medicine, School of Medicine, Ardabil University of Medical Sciences, Ardabil, Iran

**Keywords:** Gluteal, Hydatid cyst, Iran

## Abstract

Hydatid cyst caused by *Echinococcus granulosus* usually involves lung and liver but can appear in other organs. We report a 29-yr-old woman presented to Fatemi Hospital, Ardabil, Iran in 2017 with progressive painful swelling of the left gluteus which in imaging showed hydatid cyst. The cyst was successfully en blocked and the patient was discharged on albendazole treatment with no recurrence in the symptoms during the first week, first and second months after surgery follow-up and in the final visit at third months. In the endemic regions, the possibility of hydatid cysts should be considered in differential diagnosis of any cystic mass.

## Introduction

E*chinococcus granulosus* is a parasitic tape-worm responsible for hydatid cyst. Its primary hosts are dogs, sheep and cattle with humans as coincidental intermediate host (1–3). Hydatid cyst is more frequent in Africa, Europe, the Middle East, and Central and South America, Australia and Russia (2,4). In Iran this disease is endemic, especially in northwestern region (1).

Hydatid cyst mostly involves liver and lungs, but it can appear anywhere in the body (1–5). It can have variable manifestations regarding its size and the organ involved (4,5). There are few reports regarding hydatid cyst in gluteal region (2–5).

We report a case of gluteal hydatid cyst in 29-yr-old woman presented as chronic gluteal pain.

## Case presentation

A 29-yr-old woman, married, presented to Fatemi Hospital, Ardabil, Iran in 2017 with the complaint of left gluteal pain for a month following corticosteroid injection in that area. The pain was more severe in the week prior to visit caused progressive left limb lame. The patient had no history of medical disease. In physical examination, there was a swelling in the upper lateral quadrant of the left gluteal with no erythema, tenderness or warmness. Neurologic and other examinations were normal.

**Fig. 1:**
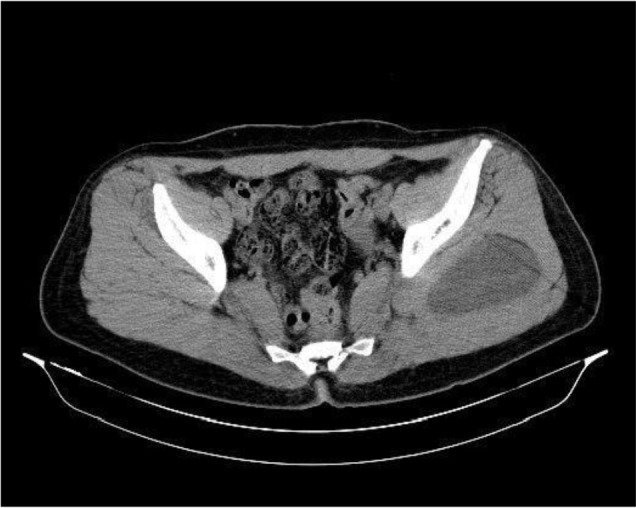
CT scan demonstrated cystic lesion in the left gluteus

**Fig. 2:**
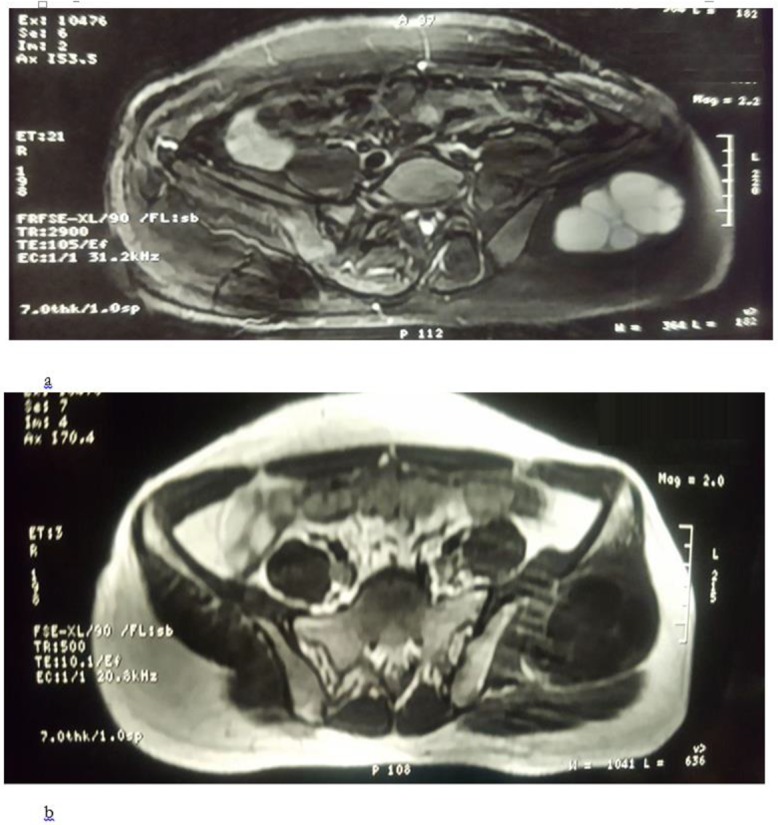
MRI showing cystic swelling in gluteus maximus in T1 (a) and T2 (b) view

Ultrasonography (US) of abdomen and chest x-ray were also normal. The US of the left gluteal showed 97*90*48 mm cystic lesion in the upper lateral quadrant with 220 cc volume with multiple septations indicative of daughter cysts. Computed tomography of pelvic showed similar findings with size of 108*76*48 mm (Fig. 1). Magnetic resonance imaging of the pelvic area also showed cystic lesion with multiple internal loculations in left deep gluteal muscle by 120*92*60 mm in dimensions suggestive for hydatid cyst (Fig. 2a, b). Moreover, unilocular cyst with 78*54*52 mm dimensions is seen in segment VII and VII liver with subcapsular extension. Other organs were otherwise normal.

**Fig. 3:**
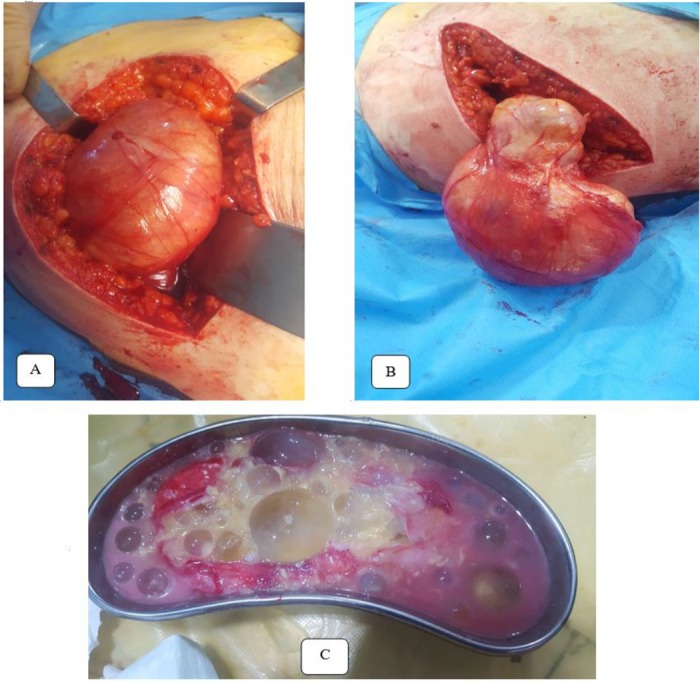
Preoperative gluteal hydatid cyst (a & b) and postoperative specimen including daughter cysts after cyst was opened

Due to the intense and persistent gluteal pain, the patients were subjected to surgical cystectomy. Albendazole 400 mg twice daily were administered a week prior to surgery. Under general anesthesia, en block surgical excision of the mass was performed with care without perforating the cyst wall (Fig. 3 a,b,c). Post-operative period was uneventful. The patient was discharged after 4 days on albendazole 400 mg twice daily for three months course. The patients were free of symptoms with no recurrence during the first week, first and second months after surgery follow-up and in the final visit at third months.

Written informed consent for patient information and images to be published was provided by the patient.

## Discussion

Hydatid cyst can appear in different organs, mostly lung and liver, but it is also reported in other areas, even in rare cases such as uterus (1) and gluteal muscle (3–5). Hydatid cyst can present with different symptoms regarding the size and site involved (1–3). Musculoskeletal hydatid cyst including gluteal cyst is very rare, and usually present with chronic painful mass in that area and usually, patients have previous history of hydatid cyst (5,6). It also may present concomitantly with hydatid cyst in other organs or be a sole presentation. In our case, the patient had painful mass in the gluteal muscle and in imaging we found hydatid cyst in the liver, as well. Therefore, in the endemic regions such as northwest of Iran, hydatid cyst should be considered as the possible differential diagnosis of any painful mass.

Hydatid cyst is usually diagnosed by history, physical examination, imaging findings and serological tests (2,4,5). In these patients, usually there is a history of animal contact (especially dogs) and living in a sheep-raising or cattle-raising rural area (7). US, CT scan and MRI are modalities that can show the cyst characteristics as well as involvement of the adjacent tissues, while in muscles MRI is more sensitive, especially evaluating the depth of the mass (3,8). All three modalities were used in our patient, and all had shown characteristics of hydatid cyst in the gluteal muscle. MRI also showed involvement of the liver in our patient.

Total surgical excision without opening the cyst is the best option for treatment of symptomatic and painful hydatid cysts, especially if the size is more than 5 cm (8,9). Medical treatment with antihelminithic drugs, such as mebendazole and albendazole, preoperatively and postoperatively should be considered besides surgery to reduce risk for local recurrence (8,10). Our patient received albendazole prior to surgery and for three months after surgery. The cyst was successfully excised with no rupture and complications.

The hydatid cyst in muscles is rare with incidence of 0.5% to 5.4% in the literature (11). However, involvement of gluteal muscle is much rare that reported in few studies (2–5,7,12–14). There are three other studies from Iran reporting the gluteal hydatid cyst including three cases from Ahvaz (15), one case from Tehran (5) and one case from Isfahan presenting as Perianal abscess (16). In all these reports, the cyst was successfully managed with surgery with no complications.

## Conclusion

Although lung and liver are the common regions for hydatid cyst, it should be considered in any patient with growing mass in any organ especially in endemic areas.
